# Critical test of isotropic periodic sum techniques with group-based cut-off schemes

**DOI:** 10.1038/s41598-018-22514-3

**Published:** 2018-03-08

**Authors:** Takuma Nozawa, Kenji Yasuoka, Kazuaki Z. Takahashi

**Affiliations:** 1 Department of Mechanical Engineering, Keio University, 3-14-1 Hiyoshi, Kohoku-ku, Yokohama, 223-8522 Japan; 2 Research Center for Computational Design of Advanced Functional Materials, National Institute of Advanced Industrial Science and Technology (AIST), Central 2, 1-1-1 Umezono, Tsukuba, Ibaraki 305-8568 Japan

## Abstract

Truncation is still chosen for many long-range intermolecular interaction calculations to efficiently compute free-boundary systems, macromolecular systems and net-charge molecular systems, for example. Advanced truncation methods have been developed for long-range intermolecular interactions. Every truncation method can be implemented as one of two basic cut-off schemes, namely either an atom-based or a group-based cut-off scheme. The former computes interactions of “atoms” inside the cut-off radius, whereas the latter computes interactions of “molecules” inside the cut-off radius. In this work, the effect of group-based cut-off is investigated for isotropic periodic sum (IPS) techniques, which are promising cut-off treatments to attain advanced accuracy for many types of molecular system. The effect of group-based cut-off is clearly different from that of atom-based cut-off, and severe artefacts are observed in some cases. However, no severe discrepancy from the Ewald sum is observed with the extended IPS techniques.

## Introduction

**Figure 1:**
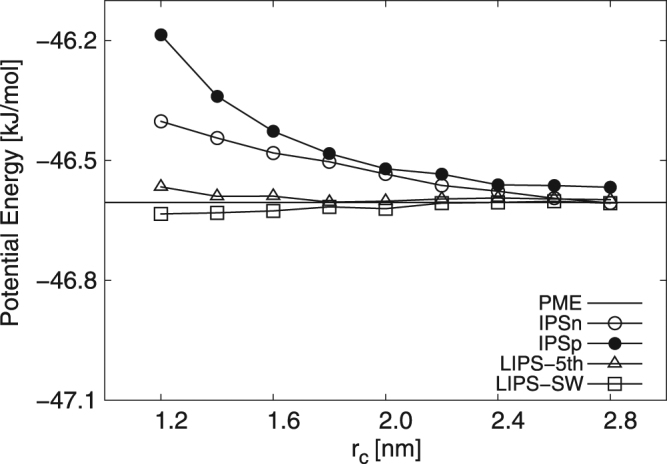
Potential energies for bulk water system calculated using the PME, IPSn, IPSp, LIPS-5th and LIPS-SW methods with group-based cut-off. IPSn overestimates potential energy with shorter cut-off and converges to PME at *r*_*c*_ = 2.6 nm; LIPS-5th and LIPS-SW converge to PME at *r*_*c*_ = 1.8 nm. By contrast, IPSp does not converge within 1.2 nm ≤ *r*_*c*_ ≤ 2.8 nm.

Molecular dynamics (MD) simulations are expected to give new insights on a molecular level into complex systems, but they incur massive computational costs. The most computationally expensive part of MD is the evaluation of the long-range interactions such as the electrostatic force. A distributed multipoles analysis gives the accurate description for spatial distribution of electric charge within a molecule and thus electrostatic structure of molecular systems^1–3^, but requires the enormous computational cost when considering electrostatic interaction calculations. Thus the electric charge of molecule is ordinary simplified using a few point charges that have constant values. However, although after the simplification, the computational cost of electrostatic interaction is still expensive to perform long-time MD simulations for large number of molecules. This cost can be reduced by employing truncation methods or lattice sum methods. The particle mesh Ewald (PME) sum is the *de facto* standard lattice sum method^4,5^, in which the long-range interaction is split into real and reciprocal space contributions. The reciprocal space can be calculated efficiently by introducing fast Fourier transforms (FFTs). The PME sum has an advanced computational efficiency, but its accuracy is guaranteed only in charge-neutral systems with periodic boundary conditions. This limitation makes it difficult to simulate some ionic systems without artificial counter ions for charge neutralization^6–9^. Thus, for net-charge systems and/or free boundary conditions, truncation has been mainly used as another approach^10–17^. Furthermore, a hierarchical tree algorithm that can be used for both free boundary and periodic boundary conditions is conceptually very similar to the truncation method, except for the hierarchical structure for the interaction calculation. Based on this similarity, several methods have been developed that combine the tree algorithm and truncation^18–21^. Switch/shift functions and Onsager’s reaction field method are some examples of typical truncation methods, but these often cause severe artefacts in various systems^22–30^. For example, switching function may induce faster decay and oscillations in the velocity autocorrelation function of ions when the switching range is too short (Fig. 2 in ref.^22^), it is well known that water transport properties are strongly influenced by shift functions (Fig. 5 in ref.^23^, Figs 1 and 5 in ref.^24^ and Fig. 2 in ref.^25^), and reaction field method with smoothing function causes unrealistic dipole-dipole correlations (Fig. 5 and 9 in ref.^26^). Therefore, the accuracy of truncation methods should be evaluated carefully. The isotropic periodic sum (IPS) method developed by Wu and Brooks^31^ is an advanced truncation method that can effectively reduce the computational cost while maintaining adequate accuracy to estimate many types of molecular system. To date, IPS techniques have been applied to many systems: solids^32^, liquids^31,33–39^, liquid–vapour interfaces^38–43^, solid–liquid interfaces^44,45^, phospholipid monolayers^46^, proteins^31,47^, combined quantum-mechanics/molecular-mechanics methods^48^, solid–liquid crystalline phase-transitions^49,50^ and constant-pH MD simulations^14–16^. Those studies suggest that IPS techniques can be applied to many macromolecular systems and that they give reasonable results. To attain better computational accuracy than that of the original IPS method, a linear-combination-based IPS (LIPS) method^38,39^ has been developed. In LIPS, several versions of pair potentials, LIPS-5th^38^, LIPS-SW^39^ and so on were designed. Our previous study showed that LIPS-SW is almost as accurate as PME with fine grid spacing (<0.1 nm) when used to estimate solid–liquid crystalline phase-transition temperatures^50^. Hence, the LIPS method is a possible way to extend the capability of the IPS technique.

Every truncation method can be implemented as one of two basic cut-off schemes, namely either an atom-based or a group-based cut-off scheme. In atom-based cut-off, only the non-bonded interactions of atoms within a given cut-off length are considered. In short, the effects of “atoms” outside the cut-off length are simply truncated. It is easily seen that the scheme could cause appreciable artefacts when considering multipole moments of polar molecules. Moreover, several truncation methods cannot be used with atom-based cut-off because of its theoretical background. For example, Onsager’s reaction field method requires that the outside of the cut-off radius be filled by a macroscopic bulk structure that consists of polar molecules. If an atom-based cut-off scheme was used for the reaction field, unexpected charged atoms would appear outside the cut-off radius. Therefore, the reaction field is normally used only with a group-based cut-off scheme. Group-based cut-off is also the standard cut-off scheme, implemented in several molecular simulation packages such as GROMACS^51–55^. In group-based cut-off, non-bonded interactions are truncated by the distance between charge groups that are defined considering charge-neutrality, functional group and residue, for example. Note that the computational accuracy of group-based cut-offs may depend on the definition of groups. The user has to define groups to attain accurate and efficient computations. Considering the charge-neutrality of groups is one of the possible ways. For example, one water molecule is proper as one group in most cases. For biological molecules, the interaction between charge-neutral groups are smoothly and continuously truncated for stabilizing the simulations^56–59^. From the perspective of molecular chemistry, this treatment is more acceptable than atom-based cut-off. Furthermore, for interaction calculations, group-based cut-off is computationally cheaper for searching interaction distances. In short, there are fewer pairs for groups than for atoms. Given its aforementioned advantages, there are many cases that group-based cut-off is more appropriate than atom-based cut-off for use with complex chemical/biological systems. However, a serious defect has been reported regarding the use of group-based cut-off for molecular systems, albeit very simple polar ones. A strange layered structure has been reported in bulk water systems^28–30^. The cause of this defect was investigated systematically, and a severe artefact around the cut-off distance was observed in the Kirkwood factor *G*_*k*_(*r*)^28,30^. This shows clearly that group-based cut-off causes serious artefacts in dipole–dipole correlations and stabilizes the anomalous layer structure in bulk water systems. This effect was also observed for switch/shift functions and the reaction field method with group-based cut-off^28,30^. Importantly, these defects were clearly reduced when using atom-based cut-off^37^. Thus we reason that group-based cut-off is more prone to erroneous behaviour than atom-based cut-off, and careful investigation is required.

As mentioned above, cut-off schemes are very important for all truncation methods. Because of how it is designed, IPS techniques are commonly used with atom-based cut-off. However, for IPS techniques to be applicable to wider varieties of simulation systems, conditions and packages, their capability with group-based cut-off should be evaluated. For example, a water molecule, which is abundant in many simulations, is treated as one charge group in most cases. Therefore, we carefully estimate herein the accuracy of IPS techniques with group-based cut-off for MD simulations of bulk water and water–vapour interfacial systems.

## Methods

### IPS methods

The fundamental IPS concept starts with dividing the potential energy *U*_*i*_ of a non-bonded interaction for particle *i* into two parts: interactions within a local region Ω_*i*_ and long-range interactions outside Ω_*i*_:

1 $${U}_{i}=\frac{1}{2}\sum _{{{\bf{r}}}_{j}\in {{\rm{\Omega }}}_{i}}[u({{\bf{r}}}_{ij})+\varphi ({{\bf{r}}}_{ij},{{\rm{\Omega }}}_{i})],$$

where ***r***_*j*_ is the position of particle *j*, ***r***_*ij*_ is the position vector from particle *i* to particle *j* and *u*(***r***_*ij*_) is the potential that describes interaction between atoms based on their distance of separation. In typical cut-off methods, *ϕ*(***r***_*ij*_, Ω_*i*_) is simply ignored or is applied to the long-range correction term based on continuum approximations. In IPS techniques, this term is expressed by a function of *r*_c_ that reflects the effect from IPS image particles^31^. To date, several different types of IPS method have been developed, for example the IPS method for non-polar systems (IPSn)^31,47^, the IPS method for polar systems (IPSp)^33^, the LIPS method with a fifth-order cut-off boundary condition (LIPS-5th)^38^ and the novel periodic reaction field method (LIPS-SW)^39^. The IPSn^31,47^ and IPSp^33^ are variations of the original IPS method, developed to calculate non-polar and polar molecular systems, respectively. However, the above two methods have difficulty to estimate homogeneous or heterogeneous polar molecular systems. The LIPS-5th^38^ and LIPS-SW^39^ are variations of the LIPS method, developed to enhance the capability of the original IPS method. These show advanced accuracy for estimating homogeneous and heterogeneous molecular systems regardless of polarity of molecules. For example, the LIPS-SW shows almost the same accuracy as the PME with fine grid spacing (<0 0.1 nm) when used to estimate solid–liquid crystalline phase transition temperatures^50^. Note that all the above characters of IPS techniques have been discovered by combinational use with the atom-based cut-off, not with the group-based cut-off. Herein, for efficient computation in GROMACS, all the IPS techniques are implemented on “tabulated interaction functions”, a basic function of GROMACS to calculate many types of interactions without editing the source code. Therefore, the observed computational costs of the methods were almost equal to each other. Other implementations can make other results on computational efficiency. Note that detailed information about the tabulated interaction functions and numerical formulation of IPS methods are given in the supporting information.

### Simulation system and conditions

MD simulations of bulk water and water–vapour interfacial systems were performed to investigate the effect of group-based cut-off scheme for the IPS techniques. The molecular-dynamics simulation package GROMACS 4.5.5 (double-precision version)^55^ was used for all simulations in this study.

The bulk system consisted of 6192 water molecules, and the extended simple point charge (SPC/E) model was used for water molecules. Three-dimensional periodic boundary conditions were used to represent the bulk water structure. The bond lengths of the atoms in each water molecule were constrained using the SHAKE method with the relative tolerance 1e-6. The Verlet velocity algorithm was used with a time step of 2 fs, which is the most common time step for water molecule with the constraint method, and known that energy drift is prevented. The simulations were performed under a constant particle-number, volume and temperature (*NVT*) ensemble with the Nosé–Hoover thermostat technique. The time constant for coupling 0.04 ps is given and the thermostat is updated every time step. The density and temperature of the system were fixed to 0.997 g/cm^3^ and 298.15 K, respectively. Non-bonded interactions were truncated with the group-based cut-off scheme. The non-bonded pair list is updated every time step to eliminate effect from the twin-range cut-off method. The cut-off radius of the Lennard-Jones (LJ) potential was set to 1.2664 nm, which is 4.0 in LJ length units for every condition. To treat the electrostatic interactions, the PME summation method, the IPSn method, the IPSp method, the LIPS-5th method and the LIPS-SW method were used. For the PME method, we chose very accurate conditions so that the interpolation was eighth order, namely a grid spacing shorter than 0.1 nm. For the IPS methods, the cut-off radius *r*_*c*_ of the electrostatic potential was changed from 1.2 to 2.8 nm in 0.2 nm increments. Note that LJ and/or electrostatic interactions (PME, IPSn, IPSp, LIPS-5th and LIPS-SW) are computed with group-based cut-off scheme in this study. The results for atom-based cut-off scheme have been given in previous researches^38,39^. The system was equilibrated for 1 ns for each condition, and then sampling calculations were performed for 1 ns to calculate equilibrium averages. To observe the dynamic characteristics of the systems, the self-diffusion coefficient *D* was calculated by the Einstein relation or the Green–Kubo formula as follows:

2 $$D=\mathop{\mathrm{lim}}\limits_{t\to \infty }\frac{1}{6t}{\langle |{{\bf{r}}}_{i}(t)-{{\bf{r}}}_{i}{\mathrm{(0)|}}^{2}\rangle }_{N},$$

where *t* is the time, ***r***_*i*_(*t*) is the position of particle *i* and <...>_*N*_ denotes the particle average. To investigate the configuration of water, the radial distribution function *g*(*r*), the distance dependence of the Kirkwood factor *G*_*k*_(*r*) and the radial orientation *h*_OO_(*r*) of the radial distribution of dipole ordering were calculated to compare the accuracy of each method. The radial distribution function *g*(*r*) and the distance dependence of the Kirkwood factor *G*_*k*_(*r*) were given by

3 $$g(r)=\frac{V}{4\pi {r}^{2}{\rm{\Delta }}rN(N-\mathrm{1)}}{\langle \sum _{i}{n}_{i}(r)\rangle }_{e},$$

4 $${G}_{k}(r)=\frac{1}{N}{\langle \sum _{i}({{\bf{u}}}_{i}\cdot \sum _{j,{r}_{ij} < r}{{\bf{u}}}_{j})\rangle }_{e},$$

where *n*_*i*_(*r*) is the number of molecules in the region between *r* and *r* + Δ*r* from molecule *i*, and ***u***_*i*_ and ***u***_*j*_ are the normalized dipole moments of molecules *i* and *j*, respectively <...>_*e*_ denotes an ensemble average at the equilibrium state. The radial orientation *h*_OO_(*r*) of the radial distribution of dipole ordering was given by

5 $${h}_{{\rm{O}}{\rm{O}}}(r)=3{g}_{{\rm{O}}{\rm{O}}}(r){\langle \cos \theta (r)\rangle }_{e},$$

where *g*_OO_(*r*) is the oxygen–oxygen radial distribution function and ${\langle \cos \theta (r)\rangle }_{e}$ is the radial orientation of the dipole, given by

6 $${\langle \cos \theta (r)\rangle }_{e}=\frac{1}{N}\langle \sum _{i}\frac{1}{{n}_{i}(r)}(\sum _{j\mathrm{=1}}^{{n}_{i}(r)}{{\bf{u}}}_{i}\cdot {{\bf{u}}}_{j})\rangle .$$

For the water–vapour interfacial system, 10,976 SPC/E water molecules were set inside a cubic simulation box of 10.1 nm on each side. The *x* and *y* axes were tangential to the interface and the *z* axis was normal to the interface. The non-bonded LJ interactions were truncated at 2.5328 nm and the cut-off radius was chosen as 5 nm for IPS methods. All other conditions were the same as those described for the bulk water case. The interfacial system had to be observed for a long time, so a production run was performed for 10 ns for each condition and a further 10 ns simulation was run to calculate equilibrium averages. To assess the accuracy for the interfacial system, the density profile ${\langle \rho (z)\rangle }_{e}$ and the electrostatic potential profile *ψ*(*z*) were calculated with respect to the surface normal (*z* axis). *ψ*(*z*) was given by double integration of the Poisson equation:

7 $$\psi (z)-\psi \mathrm{(0)=}-\frac{1}{{\varepsilon }_{0}}{\int }_{0}^{\infty }{\int }_{0}^{z^{\prime} }{\langle {\rho }_{c}(z^{\prime\prime} )\rangle }_{e}dz^{\prime\prime} dz^{\prime} ,$$

where *ε*_0_ is the vacuum permittivity and *ρ*_*c*_(*z*) is the charge density profile for the *z* direction. *ψ*(0) indicated the electrostatic potential for vacuum in liquid–vapour systems.

## Results

### Bulk water

In the following section, two cut-off schemes are described using “-atom” or “-group”, for example, IPSn method with atom-based cut-off is labelled as IPSn-atom. Figure 1 shows the potential energies calculated using the PME, IPSn, IPSp, LIPS-5th and LIPS-SW methods. The simulations were performed with several different values of the cut-off radius *r*_*c*_ to investigate the effect of *r*_*c*_ on the bulk properties. IPSn-group overestimated the potential energy with shorter cut-off and converges to PME at cut-off radii longer than *r*_*c*_ = 2.6 nm. By contrast, it has been reported that IPSn-atom underestimates the potential energy for bulk water systems^38,39^. These facts indicate that the effect of group-based cut-off on IPSn differs completely from that of atom-based cut-off. Compared to other methods, IPSp-group converges more slowly and deviates more from PME at *r*_*c*_ = 2.8 nm. This slow convergence has been reported in the case of IPSp-atom^38,39^. LIPS-5th-group and LIPS-SW-group converge to PME at cut-off radii longer than *r*_*c*_ = 1.8 nm. These results show that LIPS-5th and LIPS-SW estimate the potential energy successfully despite using group-based cut-off.

**Figure 2:**
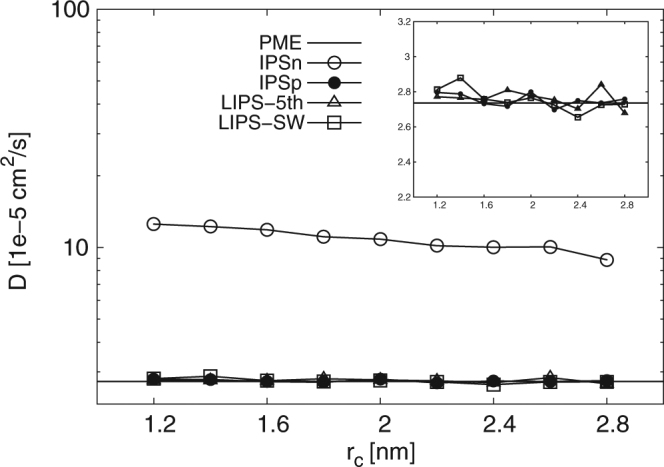
Self-diffusion coefficient *D* for bulk water system calculated using PME, IPSn, IPSp, LIPS-5th and LIPS-SW with group-based cut-off. IPSn highly overestimates *D*. By contrast, other IPS techniques agree well with PME. Inset is an enlarged view of the main graph.

Figure 2 shows the self-diffusion coefficient *D* per molecule with different values of cut-off radius for each method. IPSn-group highly overestimates *D* regardless of the cut-off distance. By contrast, IPSn-atom tends to underestimate *D* for bulk water systems^38,39^. Corresponding to the results for potential energy described above, the effects of the two cut-off schemes on IPSn clearly differ. Therefore, it is shown that the choice of cut-off scheme strongly affects not only static properties but also dynamic properties. Other IPS techniques give similar values to those of PME and successfully estimate *D* with adequate accuracy at 1.2 nm ≤ *r*_*c*_ ≤ 2.8 nm. This indicates clearly that IPSp, LIPS-5th and LIPS-SW estimate the dynamic characteristics of bulk water systems successfully despite using group-based cut-off.

**Figure 3:**
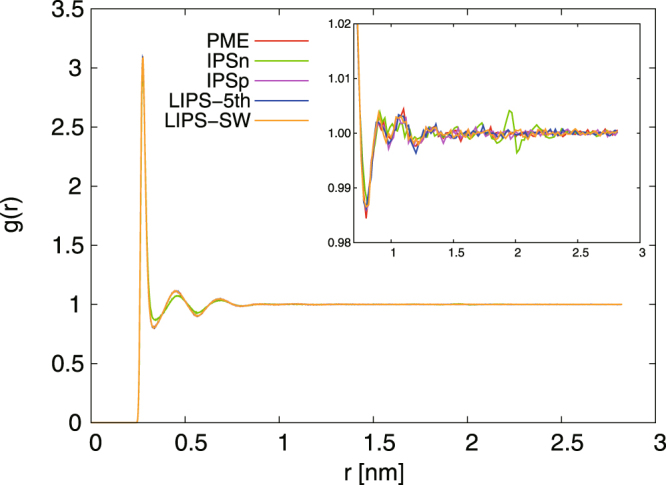
Oxygen–oxygen radial distribution function *g*(*r*) for bulk water system calculated using PME, IPSn, IPSp, LIPS-5th and LIPS-SW with group-based cut-off at *r*_*c*_ = 2.0 nm. The oxygen–oxygen *g*(*r*) of all methods except IPSn agree well with that of PME. Inset is an enlarged view of the main graph.

Figure 3 shows the oxygen–oxygen radial distribution function *g*(*r*) calculated with the PME, IPSn, IPSp, LIPS-5th and LIPS-SW methods at *r*_*c*_ = 2.0 nm. In Fig. 3 IPSn-group deviates more from PME than do the other methods. The deviation is larger than IPSn-atom^38,39^, and it hardly diminishes even when the cut-off radius is increased. By contrast, the IPSp-group, LIPS-5th-group and LIPS-SW-group forms of *g*(*r*) for agree well with the PME one. This indicates clearly that IPSp, LIPS-5th and LIPS-SW produce bulk water structures similar to that from PME despite using group-based cut-off.

**Figure 4:**
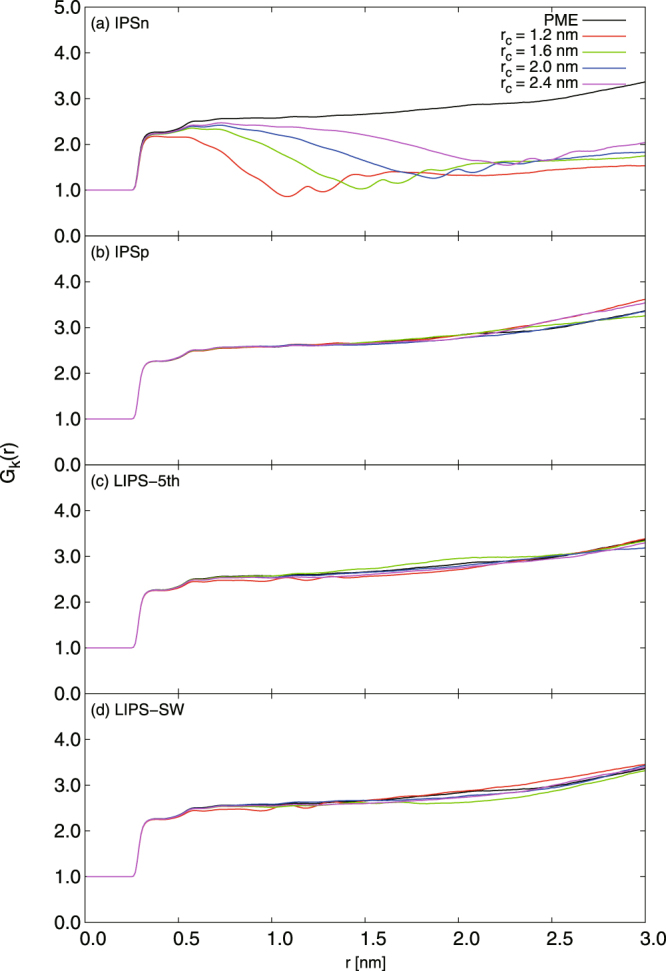
Distance-dependent Kirkwood factor *G*_*k*_(*r*) for bulk water system calculated using PME, IPSn, IPSp, LIPS-5th and LIPS-SW with group-based cut-off. For IPSn, *G*_*k*_(*r*) deviates appreciably from PME and fluctuation is observed near *r*_*c*_. The artificial configuration of *G*_*k*_(*r*) obtained with the IPSn method is not observed for IPSp, LIPS-5th and LIPS-SW.

Figure 4 shows the distance dependence of the Kirkwood factor *G*_*k*_(*r*), which indicates the dipole–dipole correlation of bulk water systems. It is well known that *G*_*k*_(*r*) is strongly affected by the choice of cut-off treatment. Thus the distance dependence of the Kirkwood factor is useful for evaluating the choice of IPS technique and cut-off scheme on the dipole–dipole correlation. In Fig. 4, *G*_*k*_(*r*) calculated with IPSn-group, IPSp-group, LIPS-5th-group and LIPS-SW-group are given and compared with that of PME. The IPSn-group *G*_*k*_(*r*) deviates appreciably from the PME one and there is also a large fluctuation near *r*_*c*_. This highlights the problem of using IPSn-group. The observed deviation is much larger than IPSn-atom, as reported previously^38,39^. The tendency of *G*_*k*_(*r*) is similar to that of the reaction field method with group-based cut-off^28^, namely that *G*_*k*_(*r*) decreases sharply near *r* = *r*_*c*_. This indicates a serious defect in dipole–dipole correlation due to the poor treatment of the cut-off boundary condition for multipoles. By contrast, it is observed that *G*_*k*_(*r*) calculated by IPSp-group, LIPS-5th-group and LIPS-SW-group is almost the same as that calculated by PME despite using group-based cut-off. The dipole–dipole correlation can be estimated reasonably using IPS techniques with group-based cut-off except when using IPSn.

**Figure 5:**
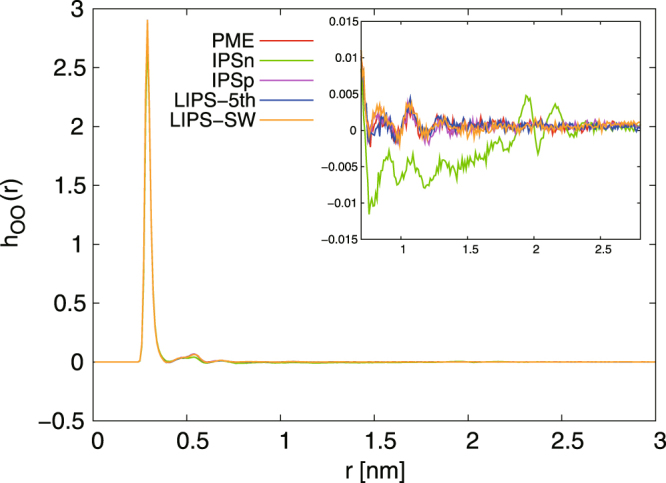
Radial distribution function *h*_OO_(*r*) of dipole ordering for bulk water system calculated using PME, IPSn, IPSp, LIPS-5th and LIPS-SW with group-based cut-off at *r*_*c*_ = 2.0 nm. All forms of *h*_OO_(*r*) agree well with the PME one except when using IPSn. Inset is an enlarged view of the main graph.

To observe dipole ordering of water molecules, the radial distribution *h*_OO_(*r*) of the dipole ordering for water molecules was calculated. Figure 5 shows *h*_OO_(*r*) calculated with the PME, IPSn, IPSp, LIPS-5th and LIPS-SW methods for *r*_*c*_ = 2.0 nm. The IPSn-group *h*_OO_(*r*) deviates more from the PME one than others. The deviation is larger than that IPSn-atom^38,39^, it hardly diminishes even when the cut-off radius is increased. By contrast, it is observed that *h*_OO_(*r*) calculated by IPSp-group, LIPS-5th-group and LIPS-SW-group is almost the same as that calculated by PME. The dipole ordering of water molecules can be estimated reasonably using IPS techniques with group-based cut-off except when using IPSn.

As shown above, using group-based cut-off strongly affects the accuracy of the IPSn method. One should note that this effect can be avoided by the practical technique for making the tabulated potential, namely the introduction of a pseudo cut-off radius for group-based cut-off. When the pseudo cut-off radius ${r}_{c}^{\ast }$ is set to be larger than the cut-off radius *r*_*c*_ for IPSn, the potential at ${r}_{c} < r < {r}_{c}^{\ast }$ is zero regardless of using group-based cut-off. Thus this treatment is numerically equal to atom-based cut-off with cut-off radius *r*_*c*_ (see supporting information).

### Water–vapour interface

**Figure 6:**
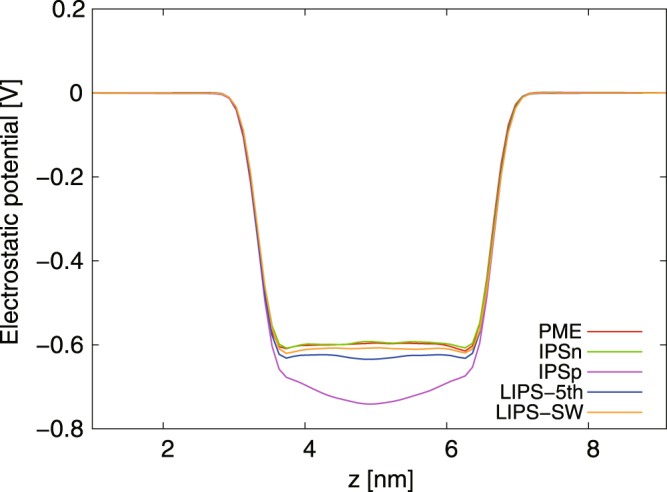
Electrostatic potential profile *ψ*(*z*) for water-vapour interfacial system calculated using PME, IPSn, IPSn, IPSp, LIPS-5th and LIPS-SW with group-based cut-off. The IPSn, LIPS-5th and LIPS-SW forms of *ψ*(*z*) agree well with the PME one, but IPSp underestimates *ψ*(*z*) appreciably at the water slab.

Figure 6 shows the electrostatic potential profile *ψ*(*z*) for the water–vapour interfacial system calculated using the PME, IPSn, IPSp, LIPS-5th and LIPS-SW methods. The IPSn-group, LIPS-5th-group and LIPS-SW-group forms of *ψ*(*z*) agree well with the PME one, while that of IPSp-group has a large discrepancy at the water slab. This discrepancy of IPSp-group is almost the same as IPSp-atom^38,39,41^. Interestingly, IPSn-group successfully estimates the physical properties of the water–vapour interfacial system but estimates the dipole-dipole correlation poorly for the bulk water system because of the differing conditions near the cut-off boundary. In our simulation, the cut-off boundaries of the water–vapour interfacial system were on the vapour phase whereas the boundaries of the bulk water system were on the water slab. The cut-off boundary has very little effect because the vapour phase contains few water molecules. Consequently, the defect of IPSn-group is concealed.

## Conclusions

The accuracy of IPS techniques with group-based cut-off was evaluated for bulk-water and water–vapour-interfacial systems. The results of using IPSn for the bulk water system highlighted the fact that the effects of atom- and group-based cut-off schemes differ clearly. The potential energy and self-diffusion coefficient were overestimated using IPSn-group but underestimated using IPSn-atom. The liquid structure calculated by IPSn-group had defects that were more serious than those calculated by IPSn-atom. For *G*_*k*_(*r*), the deviation from the results of PME was much larger than that of IPSn-atom. This indicated a serious defect in dipole–dipole correlation due to the poor treatment of the cut-off boundary condition for multipoles. Importantly, our results showed that the aforementioned problems could be avoided by using other IPS techniques. The IPSp, LIPS-5th, and LIPS-SW methods have advanced cut-off boundary treatments that were quite effective despite using group-based cut-off. Therefore, the above methods should be chosen for homogeneous polar molecular systems, but one must pay attention when using IPS techniques for heterogeneous polar molecular systems. For the water–vapour interfacial system, the calculated density and electrostatic potential profiles showed that IPSn-group is reasonably accurate without any improvement of the cut-off boundary conditions. This is because the effect of the cut-off boundary is concealed by the vapour phase, which contains few water molecules. Therefore, to estimate both homogeneous and heterogeneous polar molecular systems reasonably, the LIPS-5th and LIPS-SW perform better than other IPS techniques.

The difference between IPSn and other methods is how they treat the cut-off boundary. Because group-based cut-off requires smooth truncation at the cut-off boundary, the treatment of the higher-order derivatives of the potential energy is more critical than it is with atom-based cut-off. IPSn considers only one condition for the first derivative, therefore it may cause unphysical energy barriers at the cut-off boundary. IPSp avoids these problems by introducing a counter-charge assumption at the cut-off boundary. This makes the second and third-order differentials zero at the cut-off boundary condition, but the assumption causes poor estimation of the physical properties of interfacial water systems. In the extended IPS theory, LIPS-5th and LIPS-SW ensure that higher-order derivatives are zero on the cut-off boundary to remove unphysical energy barriers. Therefore they estimate both bulk and interfacial water systems successfully. This study shows that the aforementioned features of IPS techniques are not changed by group-based cut-off except for IPSn. One should note that the IPSm method^60^, an improved IPSn method that includes multipole interactions, may be able to overcome the above problem of IPSn.

The present work shows the robustness of the LIPS method for estimating multipolar molecular systems accurately. Therefore, LIPS methods have great potential to be combined successfully with the hierarchical tree algorithm. This may improve the previously reported IPS/tree method^20^. Combining LIPS-SW and the tree algorithm or fast multipole method may be particularly useful for computing various systems such as free boundary systems, periodic boundary systems and net-charge molecular systems.

## Electronic supplementary material

Supporting Information

## References

[1] Stone A (1981). Distributed multipole analysis, or how to describe a molecular charge distribution. Chemical Physics Letters.

[2] Stone A, Alderton M (1985). Distributed multipole analysis: methods and applications. Molecular Physics.

[3] Stone AJ (2005). Distributed multipole analysis: Stability for large basis sets. Journal of Chemical Theory and Computation.

[4] Darden T, York D, Pedersen L (1993). Particle mesh ewald: An n log (n) method for ewald sums in large systems. The Journal of Chemical Physics.

[5] Essmann U (1995). A smooth particle mesh ewald method. The Journal of Chemical Physics.

[6] Huang, Y., Chen, W., Dotson, D. L., Beckstein, O. & Shen, J. Mechanism of ph-dependent activation of the sodium-proton antiporter nhaa. *Nature Communications* **7** (2016). 10.1038/ncomms12940 PMC5059715 27708266

[7] Copie, G., Cleri, F., Blossey, R. & Lensink, M. F. On the ability of molecular dynamics simulation and continuum electrostatics to treat interfacial water molecules in protein-protein complexes. *Scientific Reports* **6** (2016). 10.1038/srep38259 PMC5131287 27905545

[8] Guan S (2017). Exploration of binding and inhibition mechanism of a small molecule inhibitor of influenza virus h1n1 hemagglutinin by molecular dynamics simulation. Scientific Reports.

[9] Borkotoky S, Meena CK, Bhalerao GM, Murali A (2017). An in-silico glimpse into the ph dependent structural changes of t7 rna polymerase: a protein with simplicity. Scientific Reports.

[10] Baptista AM, Martel PJ, Petersen SB (1997). Simulation of protein conformational freedom as a function of ph: constant-ph molecular dynamics using implicit titration. Proteins: Structure, Function, and Bioinformatics.

[11] Börjesson U, Hünenberger PH (2001). Explicit-solvent molecular dynamics simulation at constant p h: Methodology and application to small amines. The Journal of Chemical Physics.

[12] Lee MS, Salsbury FR, Brooks CL (2004). Constant-ph molecular dynamics using continuous titration coordinates. Proteins: Structure, Function, and Bioinformatics.

[13] Chen W, Shen JK (2014). Effects of system net charge and electrostatic truncation on all-atom constant ph molecular dynamics. Journal of Computational Chemistry.

[14] Wu X, Brooks BR (2015). A virtual mixture approach to the study of multistate equilibrium: Application to constant ph simulation in explicit water. PLoS Computational Biology.

[15] Lee J, Miller BT, Brooks BR (2016). Computational scheme for ph-dependent binding free energy calculation with explicit solvent. Protein Science.

[16] Wu X, Lee J, Brooks BR (2017). Origin of pka shifts of internal lysine residues in snase studied via equal-molar vmms simulations in explicit water. J. Phys. Chem. B.

[17] Socher E, Sticht H (2016). Mimicking titration experiments with md simulations: A protocol for the investigation of ph-dependent effects on proteins. Scientific Reports.

[18] Mathias G, Egwolf B, Nonella M, Tavan P (2003). A fast multipole method combined with a reaction field for long-range electrostatics in molecular dynamics simulations: The effects of truncation on the properties of water. The Journal of Chemical Physics.

[19] Mathias G, Tavan P (2004). Angular resolution and range of dipole–dipole correlations in water. The Journal of Chemical Physics.

[20] Takahashi KZ, Narumi T, Yasuoka K (2011). A combination of the tree-code and ips method to simulate large scale systems by molecular dynamics. The Journal of Chemical Physics.

[21] Lorenzen K, Schwörer M, Tröster P, Mates S, Tavan P (2012). Optimizing the accuracy and efficiency of fast hierarchical multipole expansions for md simulations. Journal of Chemical Theory and Computation.

[22] Perera L, Essmann U, Berkowitz ML (1995). Effect of the treatment of long-range forces on the dynamics of ions in aqueous solutions. The Journal of Chemical Physics.

[23] Tasaki K, McDonald S, Brady J (1993). Observations concerning the treatment of long-range interactions in molecular dynamics simulations. Journal of Computational Chemistry.

[24] Feller SE, Pastor RW, Rojnuckarin A, Bogusz S, Brooks BR (1996). Effect of electrostatic force truncation on interfacial and transport properties of water. The Journal of Physical Chemistry.

[25] Mark P, Nilsson L (2002). Structure and dynamics of liquid water with different long-range interaction truncation and temperature control methods in molecular dynamics simulations. Journal of Computational Chemistry.

[26] Alper HE, Levy RM (1989). Computer simulations of the dielectric properties of water: Studies of the simple point charge and transferrable intermolecular potential models. The Journal of Chemical Physics.

[27] Van Der Spoel D, Van Maaren PJ, Berendsen HJ (1998). A systematic study of water models for molecular simulation: derivation of water models optimized for use with a reaction field. The Journal of Chemical Physics.

[28] Van Der Spoel D, Van Maaren PJ (2006). The origin of layer structure artifacts in simulations of liquid water. Journal of Chemical Theory and Computation.

[29] Yonetani Y (2005). A severe artifact in simulation of liquid water using a long cut-off length: Appearance of a strange layer structure. Chemical Physics Letters.

[30] Yonetani Y (2006). Liquid water simulation: A critical examination of cutoff length. The Journal of Chemical Physics.

[31] Wu X, Brooks BR (2005). Isotropic periodic sum: A method for the calculation of long-range interactions. The Journal of Chemical Physics.

[32] Ojeda-May P, Pu J (2014). Assessing the accuracy of the isotropic periodic sum method through madelung energy computation. The Journal of Chemical Physics.

[33] Wu X, Brooks BR (2009). Isotropic periodic sum of electrostatic interactions for polar systems. The Journal of Chemical Physics.

[34] Takahashi K, Yasuoka K, Narumi T (2007). Cutoff radius effect of isotropic periodic sum method for transport coefficients of lennard-jones liquid. The Journal of Chemical Physics.

[35] Takahashi K, Narumi T, Yasuoka K (2010). Cutoff radius effect of the isotropic periodic sum method in homogeneous system. ii. water. The Journal of Chemical Physics.

[36] Takahashi K, Narumi T, Yasuoka K (2012). Cut-off radius effect of the isotropic periodic sum method for polar molecules in a bulk water system. Molecular Simulation.

[37] Takahashi KZ (2013). Truncation effects of shift function methods in bulk water systems. Entropy.

[38] Takahashi KZ, Narumi T, Suh D, Yasuoka K (2012). An improved isotropic periodic sum method that uses linear combinations of basis potentials. Journal of Chemical Theory and Computation.

[39] Takahashi KZ (2014). Design of a reaction field using a linear-combination-based isotropic periodic sum method. Journal of Computational Chemistry.

[40] Klauda JB, Wu X, Pastor RW, Brooks BR (2007). Long-range lennard-jones and electrostatic interactions in interfaces: application of the isotropic periodic sum method. The Journal of Physical Chemistry B.

[41] Takahashi KZ, Narumi T, Yasuoka K (2011). Cutoff radius effect of the isotropic periodic sum and wolf method in liquid–vapor interfaces of water. The Journal of Chemical Physics.

[42] Takahashi KZ (2015). An improvement of truncation method by a novel reaction field: Accurate computation for estimating methanol liquid–vapor interfacial systems. Computational Materials Science.

[43] Takahashi KZ, Yasuoka K (2015). A determination of liquid–vapour interfacial properties for methanol using a linear-combination-based isotropic periodic sum. Molecular Simulation.

[44] Nakamura H, Ohto T, Nagata Y (2013). Polarizable site charge model at liquid/solid interfaces for describing surface polarity: Application to structure and molecular dynamics of water/rutile tio2 (110) interface. Journal of Chemical Theory and Computation.

[45] Ohto T (2014). Influence of surface polarity on water dynamics at the water/rutile tio2 (110) interface. Journal of Physics. Condensed Matter: an Institute of Physics Journal.

[46] Takahashi, K. Z. Combined use of periodic reaction field and coarse-grained molecular dynamics simulations. i. phospholipid monolayer systems. *Molecular Simulation* **43**, 971 (2017).

[47] Wu X, Brooks BR (2008). Using the isotropic periodic sum method to calculate long-range interactions of heterogeneous systems. The Journal of Chemical Physics.

[48] Ojeda-May P, Pu J (2014). Isotropic periodic sum treatment of long-range electrostatic interactions in combined quantum mechanical and molecular mechanical calculations. Journal of Chemical Theory and Computation.

[49] Nozawa T, Takahashi KZ, Kameoka S, Narumi T, Yasuoka K (2015). Application of isotropic periodic sum method for 4-pentyl-4′-cyanobiphenyl liquid crystal. Molecular Simulation.

[50] Nozawa T, Takahashi KZ, Narumi T, Yasuoka K (2015). Comparison of the accuracy of periodic reaction field methods in molecular dynamics simulations of a model liquid crystal system. Journal of Computational Chemistry.

[51] Berendsen HJ, Van der Spoel D, van Drunen R (1995). Gromacs: a message-passing parallel molecular dynamics implementation. Computer Physics Communications.

[52] Lindahl E, Hess B, Van Der Spoel D (2001). Gromacs 3.0: a package for molecular simulation and trajectory analysis. Journal of Molecular Modeling.

[53] Van Der Spoel D (2005). Gromacs: fast, flexible, and free. Journal of Computational Chemistry.

[54] Hess B, Kutzner C, Van Der Spoel D, Lindahl E (2008). Gromacs 4: algorithms for highly efficient, load-balanced, and scalable molecular simulation. Journal of Chemical Theory and Computation.

[55] Pronk S (2013). Gromacs 4.5: a high-throughput and highly parallel open source molecular simulation toolkit. Bioinformatics.

[56] Brooks BR (1983). Charmm: a program for macromolecular energy, minimization, and dynamics calculations. Journal of computational chemistry.

[57] Levitt M, Sharon R (1988). Accurate simulation of protein dynamics in solution. Proceedings of the National Academy of Sciences.

[58] Kitchen DB (1990). Conserving energy during molecular dynamics simulations of water, proteins, and proteins in water. Journal of Computational Chemistry.

[59] Chiu S-W (1995). Incorporation of surface tension into molecular dynamics simulation of an interface: a fluid phase lipid bilayer membrane. Biophysical journal.

[60] Wu X, Pickard FC, Brooks BR (2016). Isotropic periodic sum for multipole interactions and a vector relation for calculation of the cartesian multipole tensor. The Journal of Chemical Physics.

