# Supplementary material for: Critical test of isotropic periodic sum techniques with group-based cut-off schemes
Source: Sci Rep. 2018 Mar 8;8:4185. doi: 10.1038/s41598-018-22514-3 (PMC5843630; doi:10.1038/s41598-018-22514-3)
Supplement: Supplementary file 1 — Supporting Information [file 41598_2018_22514_MOESM1_ESM.pdf]

Supporting Information for "Critical test of isotropic periodic sum techniques with group-based cut-off schemes"

Takuma Nozawa[1], Kenji Yasuoka[1], and Kazuaki Z. Takahashi[2,*]

[1]Department of Mechanical Engineering, Keio University, 3-14-1 Hiyoshi, Kohoku-ku, Yokohama, 223-8522, Japan
[2]Research Center for Computational Design of Advanced Functional Materials, National Institute of Advanced Industrial Science and Technology (AIST), Central 2, 1-1-1 Umezono, Tsukuba, Ibaraki 305-8568, Japan
[*]kazu.takahashi@aist.go.jp

Tabulated potential for IPS techniques

We describe how to use the IPS techniques in GROMACS. GROMACS 4.5.5 provides user-specified potential functions for non-bonded interactions given by the following equation:

$$V(r) = \frac{q_i q_j}{4\pi\varepsilon_o} f(r) + Cg(r) + Ah(r), \tag{1}$$

where the first, second and third terms represent the electrostatic, dispersion and repulsion interactions, respectively. $f(r)$, $g(r)$ and $h(r)$ are user-defined functions that are specified in the user's table file. The table file should contain r and the values of $f(r)$, $g(r)$ and $h(r)$ and their derivatives at r. For example, the normal Lennard–Jones and Coulomb potentials are given as follows:

$$f(r) = \frac{1}{r}, \qquad g(r) = -\frac{1}{r^6}, \qquad h(r) = \frac{1}{r^{12}}. \tag{2}$$

In the same way, the user can define the IPS potentials by replacing $f(r)$ with the following functions:

$$f_{\text{IPSn}}(r, r_c) = 1 + \frac{4}{13}\left(\frac{r}{r_c}\right)^3 + \frac{1}{26}\left(\frac{r}{r_c}\right)^5 + \frac{1}{26}\left(\frac{r}{r_c}\right)^7, \tag{3}$$

$$f_{\text{IPSp}}(r, r_c) = 1 + \frac{35}{16}\left(\frac{r}{r_c}\right)^3 - \frac{21}{16}\left(\frac{r}{r_c}\right)^5 + \frac{5}{16}\left(\frac{r}{r_c}\right)^7, \tag{4}$$

$$f_{\text{LIPS-5th}}(r, r_c) = \frac{1}{r} - \frac{1}{2r_c}\sum_{k_f=1}^{n_f} \alpha_{k_f}(r/r_c)^{2k_f}, \tag{5}$$

$$f_{\text{LIPS-SW}}(r, r_c) = \frac{1}{r} - \frac{1}{2r_c}\left[(r/r_c)^2 - \alpha^2\right]^3 \sum_{k_f=1}^{n_f} \alpha_{k_f}(r/r_c)^{2k_f}, \tag{6}$$

where r_c is the cut-off distance for the IPS potential and the coefficients for Eqs. (5) and (6) are given in Tables 1 and 2, respectively. See the manual of GROMACS 4.5.5. for detailed information about the usage of tabulated potentials in GROMACS.

Improvement of accuracy with longer cut-off condition

Group-based cut-off strongly affects the accuracy of the IPSn method. This effect can be avoided by introducing a pseudo cut-off radius for group-based cut-off. When the pseudo cut-off radius r_c^* is set to be larger than the cut-off radius r_c for IPSn, the potential at $r_c < r < r_c^*$ is zero regardless of using group-based cut-off. Thus this treatment is numerically equal to atom-based cut-off with cut-off radius r_c. For comparison, we performed molecular dynamics (MD) simulations of the bulk water system under the same conditions as those in the main paper except that the cut-off distance is chosen as $r_c^* = r_c + 0.2$ nm for the non-bonded Coulomb potential.

Figure 1 shows the potential energies calculated using the PME, IPSn, IPSp, LIPS-5th and LIPS-SW methods (see the main paper for the definitions of these acronyms). The simulations were performed with several different values of the cut-off radius r_c to investigate the effects on the bulk properties. The IPSn, LIPS-5th and LIPS-SW methods converge to the PME one at r_c =

Table 1. Numerical fitting parameters for LIPS-5th Coulomb potential.

n_f	k_f	a_{k_f}
12	1	−0.60102948970315
	2	−0.129545033656405
	3	−0.0345281587231329
	4	0.0739341439476294
	5	−1.37699830398588
	6	13.6315588578579
	7	−70.3070554901045
	8	140.340476904515
	9	−121.490128988349
	10	35.1421425921193
	11	9.08814424553541
	12	−5.31588924671815

Table 2. Numerical fitting parameters for LIPS-SW Coulomb potential.

n_f	k_f	a_{k_f}
9	1	0.0125143224110408
	2	−0.603493863454666
	3	11.7355819865242
	4	−96.296895305654
	5	216.649868508398
	6	−197.409191110696
	7	59.9544311773618
	8	13.9564907382725
	9	−8.66620089071555

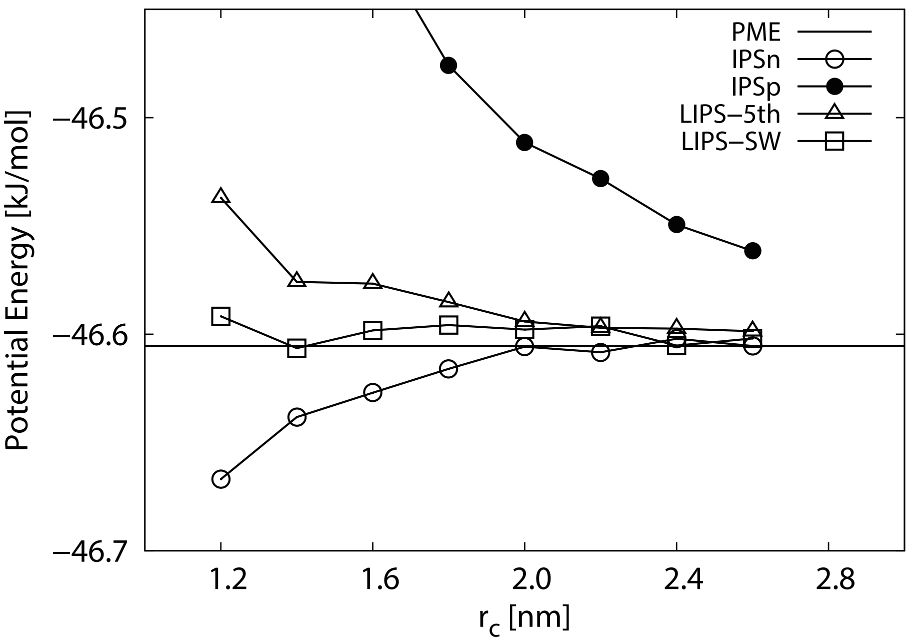

Figure 1. Potential energies for bulk water system calculated using PME, IPSn, IPSp, LIPS-5th and LIPS-SW with group-based cut-off. IPSn, LIPS-5th and LIPS-SW converge to PME at r_c = 2.0 nm, whereas IPSp does not converge within 1.2 nm $\leq r_c \leq$ 2.8 nm. This trend agrees well with the case of atom-based cut-off.

2.0 nm. By contrast, the IPSp method does not converge within 1.2 nm $\leq r_c \leq$ 2.8 nm. The potential energies agree well with those obtained with atom-based cut-off[1], clearly indicating that the accuracy of IPSn is improved considerably by adopting a longer cut-off distance.

To assess the dynamic characteristics of water molecules, the self-diffusion coefficient D was calculated. Figure 2 shows the self-diffusion coefficient per molecule calculated using the PME, IPSn, IPSp, LIPS-5th and LIPS-SW methods with longer cut-off distance. IPSn converges to PME at r_c = 2.0 nm, and the other methods successfully estimate D with adequate accuracy at 1.2 nm $\leq r_c \leq$ 2.8 nm. The values of D also agree well with those obtained using atom-based cut-off[1], clearly indicating that the accuracy of IPSn is improved considerably by adopting a longer cut-off distance.

To observe the structure of water molecules, the radial distribution function $g(r)$ was calculated. Figure 3 shows the oxygen–oxygen radial distribution function calculated with the PME, IPSn, IPSp, LIPS-5th and LIPS-SW methods at $r_c = 2.0$ nm. The deviation of IPSn from that of PME is decreased considerably but there is still a small fluctuation near the cut-off distance r_c. The IPSp, LIPS-5th and LIPS-SW methods give very similar results to those of PME. This trend agrees well with that obtained using atom-based cut-off.

Figure 4 shows the distance dependence of the Kirkwood factor $G_k(r)$, which indicates the dipole–dipole correlation of bulk water systems. With the IPSn method, $G_k(r)$ still fluctuates near the cut-off distance r_c but the deviation from that of PME is decreased considerably. The IPSp, LIPS-5th and LIPS-SW methods give very similar results to those of PME. This trend agrees well with that obtained using atom-based cut-off.

To observe dipole ordering of water molecules, the radial distribution $h_{OO}(r)$ of the dipole ordering for water molecules was calculated. Figure 5 shows $h_{OO}(r)$ calculated with the PME, IPSn, IPSp, LIPS-5th and LIPS-SW methods for r_c = 2.0 nm. The deviation of IPSn is decreased considerably compared to that of PME but there is still a small fluctuation near the cut-off distance r_c. The IPSp, LIPS-5th and LIPS-SW methods give very similar results to those of PME. This trend agrees well with that obtained using atom-based cut-off.

Additional results of water–vapour interfacial systems

Figure 6 shows the density profiles for the water–vapour interfacial system calculated using PME, IPSn, IPSp, LIPS-5th and LIPS-SW methods. Note that the simulation conditions are same as those in the main paper. The results indicate that all IPS techniques successfully provide density profiles similar to the PME one despite using group-based cut-off.

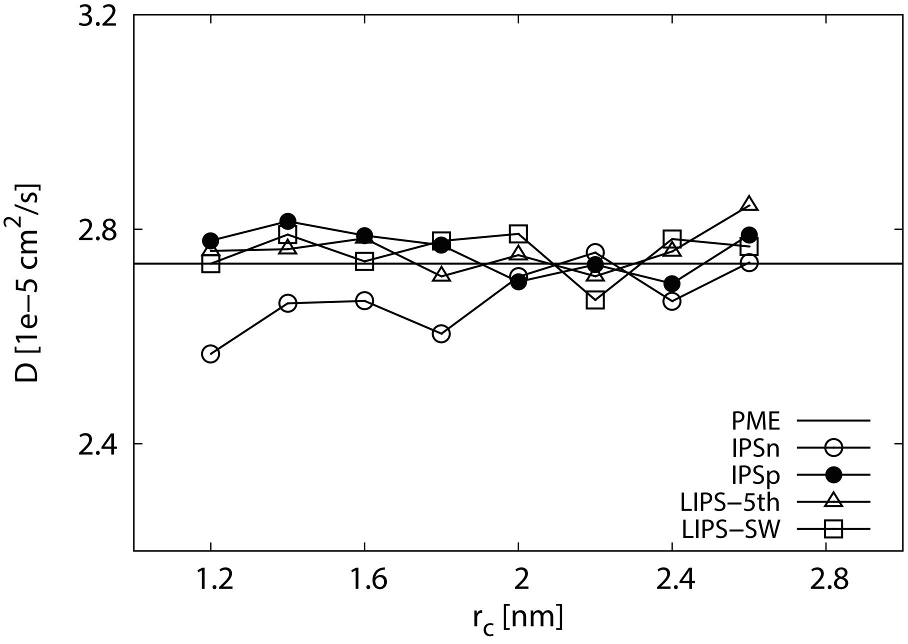

Figure 2. Self-diffusion coefficient D for bulk water system calculated using PME, IPSn, IPSp, LIPS-5th and LIPS-SW with group-based cut-off. The IPSn D converges to the PME one at r_c = 2.0 nm. The other methods successfully estimate D with adequate accuracy at 1.2 nm $\leq r_c \leq$ 2.8 nm. This trend agrees well with that obtained using atom-based cut-off.

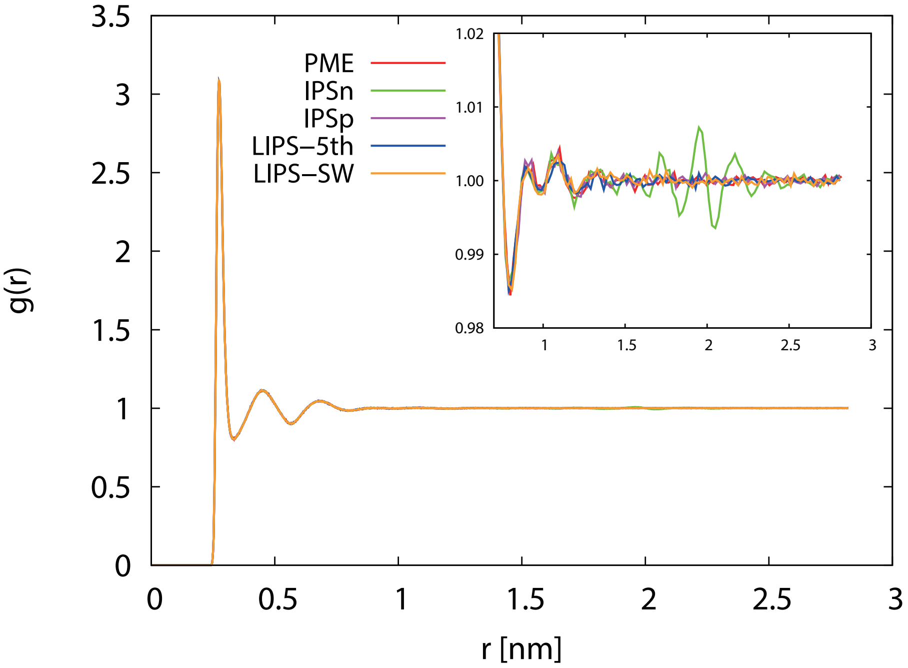

Figure 3. Oxygen–oxygen radial distribution function $g(r)$ for bulk water system calculated using PME, IPSn, IPSp, LIPS-5th and LIPS-SW with group-based cut-off at r_c = 2.0 nm. The deviation of IPSn is decreased considerably but there is still a small fluctuation near the cut-off distance r_c. IPSp, LIPS-5th and LIPS-SW give very similar results to those of PME. This trend agrees well with that obtained using atom-based cut-off.

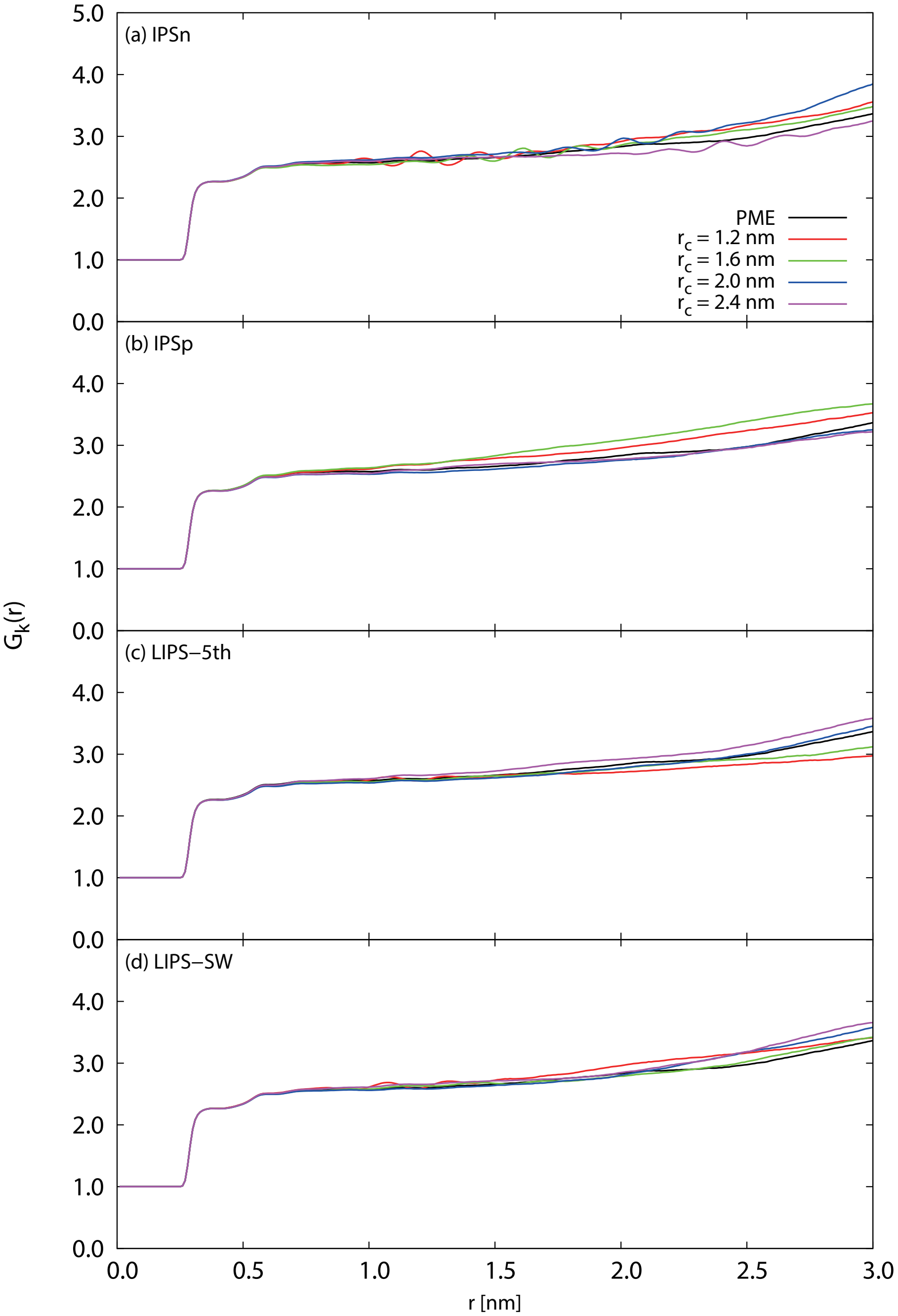

Figure 4. Distance-dependent Kirkwood factor $G_k(r)$ for bulk water system calculated using PME, IPSn, IPSp, LIPS-5th and LIPS-SW with group-based cut-off. With the IPSn method, $G_k(r)$ still fluctuates but the deviation is decreased considerably. IPSp, LIPS-5th and LIPS-SW give very similar results to those of PME. This trend agrees well with that obtained using atom-based cut-off.

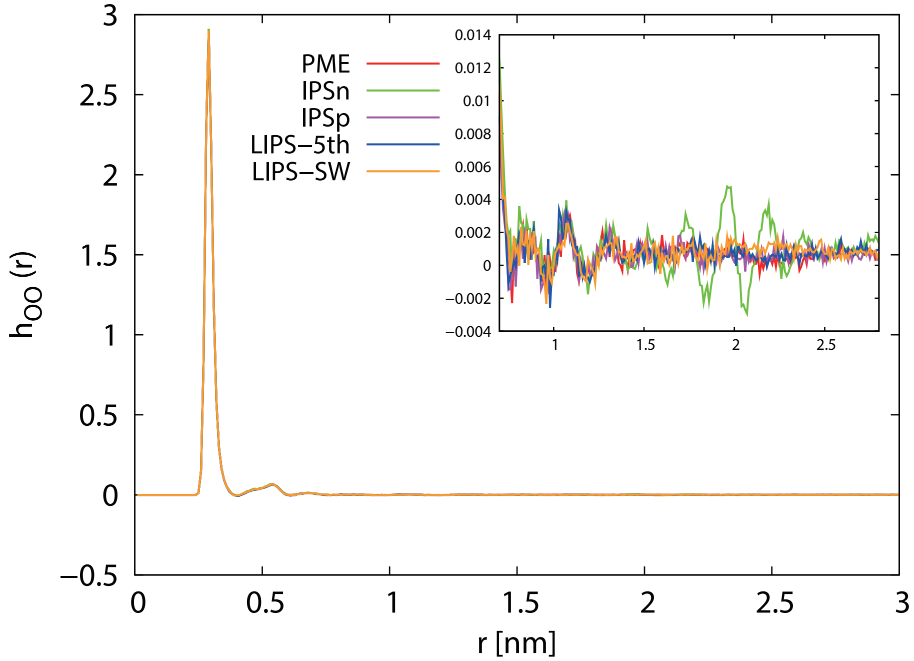

Figure 5. Radial distribution function $h_{OO}(r)$ of dipole ordering for bulk water system calculated using PME, IPSn, IPSp, LIPS-5th and LIPS-SW with group-based cut-off at r_c = 2.0 nm. The deviation of IPSn is decreased considerably but there is still a small fluctuation near the cut-off distance r_c. IPSp, LIPS-5th and LIPS-SW give very similar results to those of PME. This trend agrees well with that obtained using atom-based cut-off.

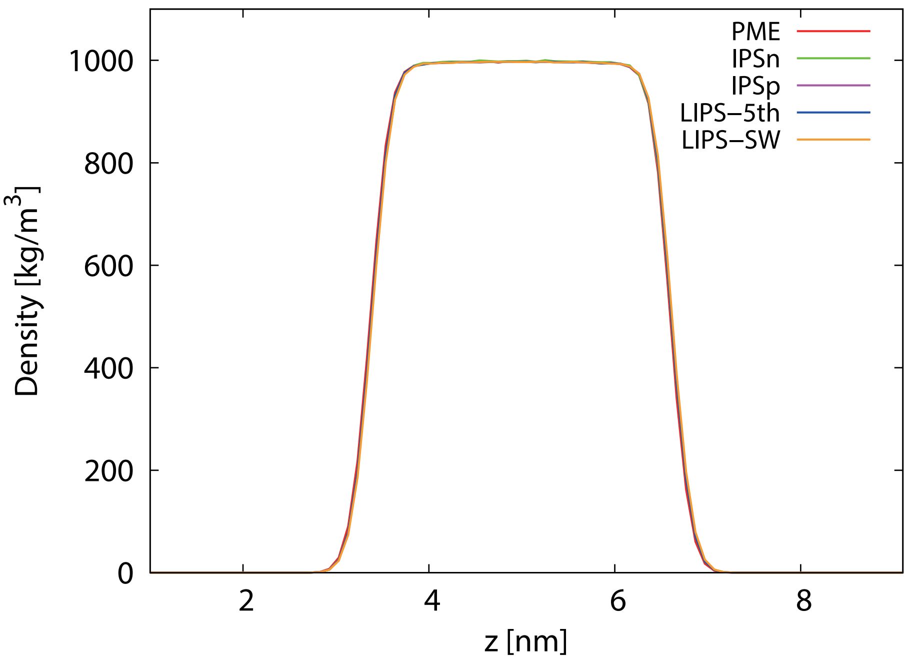

Figure 6. Density profiles for water–vapour interfacial system calculated using PME, IPSn, IPSn, IPSp, LIPS-5th and LIPS-SW with group-based cut-off. The results for all IPS techniques are similar to those for PME.

References

1. Takahashi, K. Z. Design of a reaction field using a linear-combination-based isotropic periodic sum method. *J. Comput. Chem.* **35**, 865 (2014).
